# Hotspots of Floating Plastic Particles across the North Pacific Ocean

**DOI:** 10.1021/acs.est.3c05039

**Published:** 2024-02-23

**Authors:** Robby Rynek, Mine B. Tekman, Christoph Rummel, Melanie Bergmann, Stephan Wagner, Annika Jahnke, Thorsten Reemtsma

**Affiliations:** † Department of Analytical Chemistry, Helmholtz Centre for Environmental Research − UFZ, 04318 Leipzig, Germany; ‡ Alfred-Wegener-Institut, Helmholtz-Zentrum für Polar- und Meeresforschung, 27570 Bremerhaven, Germany; § Department of Natural and Mathematical Sciences, Faculty of Engineering, Ozyegin University, 34794 Istanbul, Turkey; ∥ Department of Bioanalytical Ecotoxicology, Helmholtz-Centre for Environmental Research − UFZ, 04318 Leipzig, Germany; ⊥ Department of Exposure Science, Helmholtz-Centre for Environmental Research − UFZ, 04318 Leipzig, Germany; # Institute for Environmental Research, RWTH Aachen University, 52047 Aachen, Germany; ∇ Institute of Analytical Chemistry, University of Leipzig, Linnéstrasse 3, 04103 Leipzig, Germany

**Keywords:** marine pollution, marine litter, anthropogenic debris, microplastic, neuston catamaran, spatial distribution, ATR-FT-IR, FT-IR imaging

## Abstract

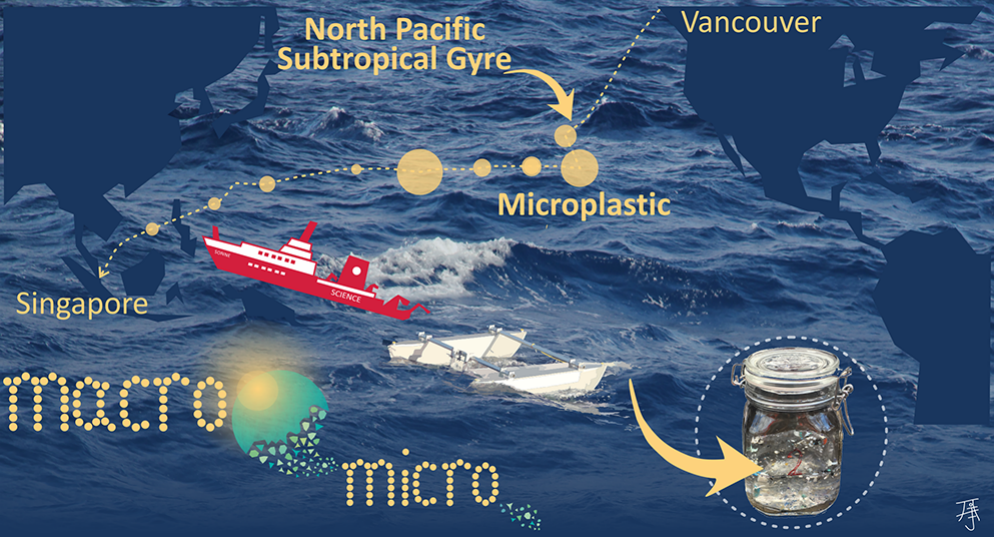

The pollution of the marine environment with plastic debris is expected to increase, where ocean currents and winds cause their accumulation in convergence zones like the North Pacific Subtropical Gyre (NPSG). Surface-floating plastic (>330 μm) was collected in the North Pacific Ocean between Vancouver (Canada) and Singapore using a neuston catamaran and identified by Fourier-transform infrared spectroscopy (FT-IR). Baseline concentrations of 41,600–102,700 items km^–2^ were found, dominated by polyethylene and polypropylene. Higher concentrations (factors 4–10) of plastic items occurred not only in the NPSG (452,800 items km^–2^) but also in a second area, the Papaha̅naumokua̅kea Marine National Monument (PMNM, 285,200 items km^–2^). This second maximum was neither reported previously nor predicted by the applied ocean current model. Visual observations of floating debris (>5 cm; 8–2565 items km^–2^ and 34–4941 items km^–2^ including smaller “white bits”) yielded similar patterns of baseline pollution (34–3265 items km^–2^) and elevated concentrations of plastic debris in the NPSG (67–4941 items km^–2^) and the PMNM (295–3748 items km^–2^). These findings suggest that ocean currents are not the only factor provoking plastic debris accumulation in the ocean. Visual observations may be useful to increase our knowledge of large-scale (micro)plastic pollution in the global oceans.

## Abstract 2

Two independent approaches revealed baseline contamination of floating plastic items across the North Pacific Ocean, with hotspots in the North Pacific Subtropical Gyre and a marine-protected World Heritage Site.

## Introduction

Plastic debris is a ubiquitous contaminant with increasing concentrations in the open ocean^1^ such that the pollution of the environment with plastic is considered a planetary boundary threat.^2−5^ Since the beginning of industrial plastic production in the 1950s, it has grown by about 8% per year and exceeded 400 Mt in 2022.^6−8^ Plastic waste can either be transported into the oceans by rivers,^9^ enter the sea from coastal regions^10^ and ships,^11^ or be generated directly during use, e.g., through fishing activities.^12^ In the environment, weathering processes promote the fragmentation of larger plastic debris into smaller plastic items.^13^ Assuming a business-as-usual scenario, the annual input of plastic waste into the aquatic environment could increase from 19–23 million metric tons (MMT) in 2016 to 35–90 MMT in 2030, leading to an ever-increasing pollution of the oceans.^14^ Despite increasing production volumes and continued inputs of plastic waste into the aquatic environment, studies indicate the concentration of plastics in the world’s oceans to be at a stable level in recent years but showing an increase of plastic amounts in remote regions where the accumulation is poorly reversible like in the open ocean, remote islands, polar regions, and the deep sea.^15,16^ Since the available data are patchy with poor comparability of studies due to different nonstandardized methods of sampling, analysis, and data reporting, it is still difficult to robustly constrain the overall extent of large-scale plastic pollution in the oceans. However, there is growing concern about the adverse ecological,^17^ economic,^18^ and social effects^19^ of plastic waste in the marine environment.

More than half of the plastic mass ever produced has an initial density lower than seawater and is hence expected to float on the ocean surface until it is altered by weathering, fouling, or ballasting processes.^6,20^ Ocean currents and prevailing winds cause horizontal transport and an accumulation of these buoyant plastic particles in ocean gyres.^21^ The largest known accumulation zone for floating plastic debris is the so-called “Great Pacific Garbage Patch” (GPGP) in the area of the North Pacific Subtropical Gyre (NPSG) between the western U.S. coast and Hawaii.^22^ While most studies conducted in the North Pacific Ocean have focused on this accumulation area and coastal regions of Asia and North America, little is known about the extent of plastic pollution across the North Pacific Ocean. Furthermore, some published data about the pollution of the marine environment are based on visual observations and remote sensing from ships or aircrafts, targeting larger plastic debris in the size range from several centimeters to meters, hence lacking information about the distribution of small plastic and microplastic particles for these studies.

This work evaluates the large-scale distribution of floating plastic items >330 μm sampled from nine stations along the track of a dedicated scientific expedition crossing the North Pacific Ocean between Vancouver (Canada) and Singapore. The goals were 3-fold: (i) to get an impression of the baseline concentrations of floating (micro)plastic items along that transect across the North Pacific Ocean compared to the previously described hotspot area in the NPSG; (ii) to investigate spatial patterns of size composition to determine if the increasing distance from coastal source regions is correlated with larger degrees of weathering-induced fragmentation, affecting the particle size distribution, and (iii) to compare (micro)plastic concentrations to a data set collected in parallel through visual observations of floating macrodebris to assess if the concentrations of macrodebris (>5 cm) can be used as a proxy for small plastic items (>330 μm).

## Materials and Methods

### Sampling Procedure

**Figure 1:**
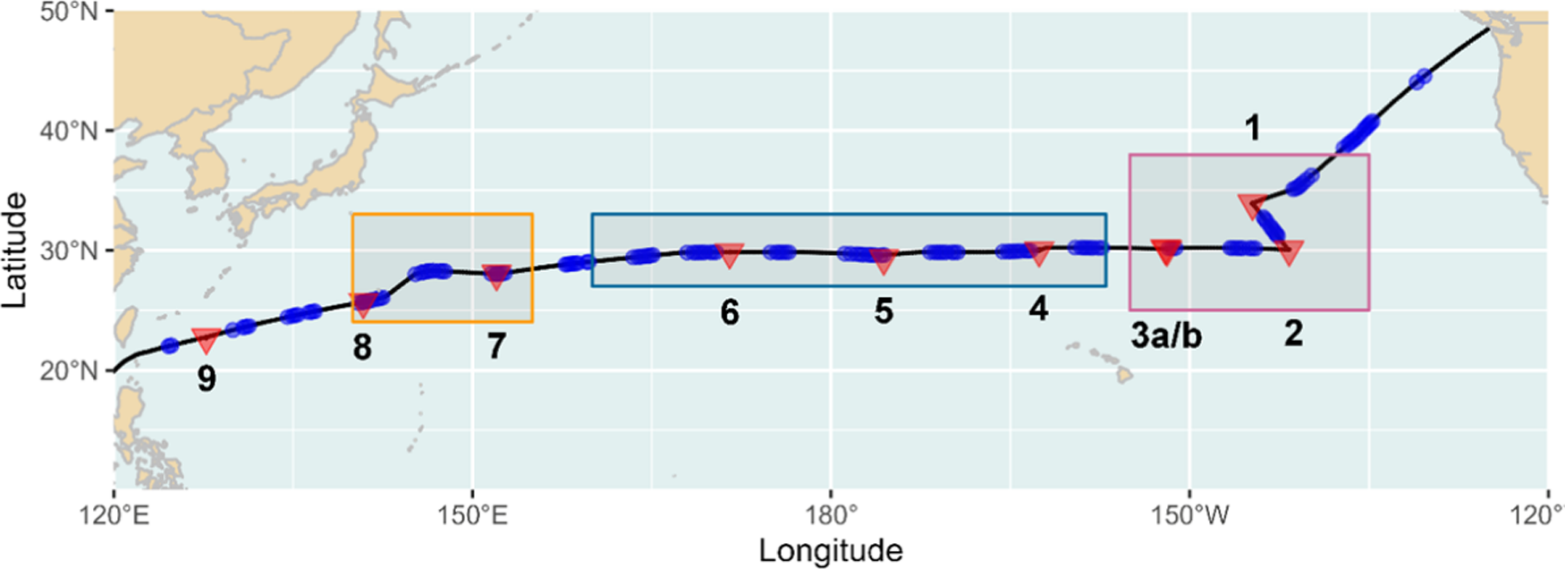
Map of the North Pacific Ocean with the cruise track of SO268/3 (black line) and model-projected areas of the so-called Eastern Garbage Patch (red rectangle), Subtropical Convergence Zone (blue rectangle), and the so-called Western Garbage Patch (orange rectangle). Red triangles represent the positions of catamaran samplings; blue dots represent the positions of visual observations.

Samples were collected during the expedition SO268/3 of the research vessel (RV) SONNE between Vancouver (BC, Canada) and Singapore from May to July 2019 (Figure 1). All sampling-related information is provided in the Supporting Information (Table S1). Sampling locations were selected based on the Surface Currents from Diagnostic (SCUD) model^23^ to cover potentially highly contaminated areas (stations 1–3) and presumably less contaminated, less studied, and more remote areas in the open ocean (stations 4–9).^21^ The model projected the highest concentration of plastic debris in the center of the so-called Eastern Garbage Patch (Figure 1, red rectangle) between 30–35°N and 135–145°W. Because of the surrounding North Pacific Current in the North, the California Current in the East, the North Equatorial Current in the South, and the Kuroshio Current in the West, elevated plastic concentrations are generally expected westward of the prime accumulation zone (Figure 1, red rectangle) surrounding the 30°N latitude, where the so-called Western Garbage Patch (Figure 1, orange rectangle) and the Subtropical Convergence Zone (Figure 1, blue rectangle) are located.

The sea surface was sampled by a neuston catamaran (Figure S1, HYDRO-BIOS Apparatebau GmbH, Germany), which is particularly well-suited to higher wave conditions.^24^ It was equipped with a net having a mesh size of 330 μm and a length of 4 m. The net opening of 30 cm × 15 cm was equipped with a mechanical flow meter (HYDRO-BIOS), whose measuring wheel is turned by the motion of water and slows and eventually stops if the net moves out of the water. The catamaran was towed alongside the ship with a rope length of 40 m for an average time of 45 min at 4 kn (3 kn at station 1), resulting in sampled surface areas between 1081 and 1791 m^2^ and volumes between 162 and 268 m^3^. Sampling was conducted at nine stations along the cruise track (Figure 1, red triangles). At station 3, duplicate samples (referred to as a and b) were taken sequentially to assess the small-scale variability of plastic pollution.

All sample handling was conducted on deck. After each deployment, the neuston net was dismantled from the mouth opening and immediately covered with aluminum foil to avoid contamination. The collected particles, lines, and filaments (hereafter referred to as plastic items) in the body of the net were washed thoroughly with excessive seawater from the outside downward into the sample bucket. Once no apparent material remained in the net, the bucket in the cod end was detached, the sample was directly transferred into a precleaned 1 L wireframe glass jar (Flaschenland GmbH, Germany), and the jar was sealed immediately to avoid contamination. The open net was then rinsed thoroughly to ensure a clean net for the next deployment. Between stations, the catamaran net was submerged in an aqueous detergent solution for at least 3 h, afterward rinsed from the outside with seawater, and wrapped in aluminum foil until the next deployment. Procedural blanks were taken by rinsing the cleaned catamaran net with seawater from the outside in the identical way as samples were processed, followed by transferring the residue in the cod end of the net into glass containers. Samples and blanks were transported frozen to our institute and stored at −20 °C until processing.

### Sample Preparation

After defrosting, the samples and blanks were first filtered through a stainless steel sieve with a mesh size of 2 mm (Retsch GmbH, Germany), and the retained particles were rinsed thoroughly with 200 mL of ultrapure, particle-free Milli-Q water (Milli-Q Direct 8, Merck KGaA, Germany). Putative plastic items from the >2 mm fraction were sorted, stored individually in 1.5 mL centrifuge tubes (Eppendorf SE, Germany), and characterized visually regarding size, shape, and color. Furthermore, plastic items showing signs of biological growth on their surface were classified as biofouled.

The remaining sample suspensions with particles <2 mm were rinsed into stainless steel reactors semienclosed with 10 μm stainless steel filters (Haver & Boecker OHG, Oelde, Germany).^25^ This sample fraction was treated according to an enzymatic purification protocol, which has previously been shown not to alter the size and number of particles (Text S1).^26^ After the last step of the purification protocol, the remaining particles on the 10 μm steel filters and the inner surface of the reactors were thoroughly rinsed through a 500 μm stainless steel sieve (Retsch GmbH, Germany) into glass beakers for further size fractionation. Putative plastic items >500 μm were visually extracted with tweezers and stored in individual centrifuge tubes. The remaining fraction <500 μm was filtered onto aluminum oxide filters (Anodisc, 0.2 μm pore size, 25 mm diameter, Whatman PLC, U.K.), placed in Petri dishes, and dried overnight at 50 °C in an oven (Heraeus T6120, Thermo Fisher Scientific).

### ATR-FT-IR Measurements

All manually selected putative plastic items ≥500 μm were analyzed using a Cary 620 FT-IR microscope equipped with a Ge ATR crystal connected to a 15× magnification objective (Agilent Technologies). Measurements were performed with 16 scans between 400 and 4000 cm^–1^ and a spectral resolution of 4 cm^–1^. Spectra were exported from Resolutions Pro (v. 5.4.1.3412, Agilent Technologies), analyzed using the SiMPle software (v. 1.01, database version 1.0.1), and assigned to the respective polymer.^27^ Raw spectra and first derivatives were used equally weighted for spectral comparison in the ranges of 3300–2700 and 1900–1250 cm^–1^, and the inspected pieces were assigned to the polymer with the highest ranked spectral match with a minimum value of 0.5.

### FT-IR Imaging

Imaging analysis of filter samples containing the putative plastic items in the range of 330–500 μm was conducted with a Cary 620 FT-IR microscope equipped with a 15× objective and a 128 × 128 Lancer MCT FPA detector array (Agilent Technologies) in transmission mode. The dried Anodisc filters with the samples were placed on a steel filter holder and covered with an IR-transparent BaF_2_ window (Korth Kristalle GmbH, Germany). FT-IR measurements of the whole filter were carried out with 8 scans in the spectral range of 3800–1250 cm^–1^, a spectral resolution of 8 cm^–1^, and a spatial resolution of 5.5 × 5.5 μm^2^ per spectrum. Imaging data sets were analyzed using the machine-learning algorithms of the software Microplastics Finder v. 4.09 (Purency GmbH, Austria) to identify and count plastic items and determine their size as minimum Feret diameter based on the two-dimensional (2D) FT-IR imaging data.^28^ Postprocessing was done by excluding the polypropylene (PP) support ring of the Anodisc filter from the data set. Additionally, assignments with less than four pixels or with relevance and similarity scores below 0.6 were removed. Outlines of all detected plastic items were cross-validated with photos of the analyzed filters and corrected manually if necessary.

### Quality Assurance and Quality Control

To minimize ship-borne contamination, the samples were extracted from the net’s cod end into airtight containers on the ship’s deck immediately after hauling, sealed, stored, and shipped frozen to our laboratories. All sample preparation steps, except the enzymatic/oxidative purification in the semienclosed reactor vessels, were conducted under a safety workbench (HeraSafe HS9, Kendro Laboratory Products GmbH, Germany) to minimize airborne contamination. Laboratory equipment was thoroughly rinsed with Milli-Q water and prefiltered ethanol (96%, CHEMSOLUTE, Germany) and covered with aluminum foil until use. The use of plastic material during sampling and processing was avoided wherever possible to minimize contamination. Plastic materials used during sampling and analysis were polyamide (PA, neuston net), poly(vinyl chloride) (PVC, cod end), poly(tetrafluoroethylene) (PTFE, sealing of reactors), ethylene tetrafluoroethylene (ETFE, squeeze bottle), and PP (Anodisc support ring). Negative controls were generated by analyzing two procedural blank samples after identical treatment as the environmental samples. Possible loss of particles during the purification procedure was evaluated by treating three artificial samples containing 20 polyethylene particles (PE, fragments, <1 mm) each identically as the environmental samples. The influence of the prevailing sea state on the efficiency of the net sampling was evaluated using a simple linear regression between the sea state (Beaufort scale) and the log 10 of plastic item concentrations.^29^

### Calculation of Concentrations from Catamaran Samples

Area-normalized concentrations of plastic items *c*_A_ in items km^–2^ (Table S1) were calculated using the number of detected plastic items *N* and the sampled sea surface area *A* (eq 1). The sampled area was calculated by using the tow distance *D* measured by the flow meter and the width of the catamaran net opening *w*_cat_. Additional information about the performance of the used flow meter at different sampling conditions can be found in the Supporting Information (Text S2 and Figures S2 and S3)
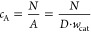
1

Volume-based concentrations of plastic items *c*_V_ in items m^–3^ (Table S1) were calculated by adding the height of the net opening *h*_Cat_ to the calculation in eq 2
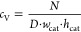
2

As the neuston net was not fully submerged, we estimated the active height of the net to be 50% of the height of the opening, which was 7.5 cm. Due to the catamaran’s up- and downward movement, this estimate may be biased. Therefore, only area-based concentrations are further discussed in the main manuscript and volume-based concentrations are reported in the Supporting Information.

The applied surface water sampling technique may underestimate the measured plastic item concentration since high sea states driven by local wind-mixing are known to increase vertical wind-induced mixing.^30^ To improve the comparability of our data between stations and with published data sets, the observed concentrations were corrected for wind-induced vertical mixing of the upper water column. For this purpose, a one-dimensional model was used to predict the vertical distribution of floating plastic items and calculate depth-integrated concentrations for the upper 5 m of the water column (Text S2).^22,30,31^ As the vertical transport depends on the sea state, particle type, and particle size, plastic items from catamaran samples were classified regarding the type and size (Table S2), and concentrations *c*_A_ of each class were calculated for every sample (Table S3). Wind-mixing-corrected concentrations *c*_i_ in N km^–2^ of plastic items from different sizes and type classes in the upper water column were calculated using the observed area concentration *c*_A_ (Table S3) and correction factors *F* (Table S4) to level out the influence of different sea states during sampling according to eq 3(22,30,31)
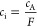
3

Wind-mixing-corrected concentrations for every type and size class and total concentrations of every sample can be found in the SI (Table S5).

### Comparison of Catamaran Sampling Data (Small Plastic Items) to Visual Observations (Macroplastic)

Visual observations were conducted whenever the ship was in transit to quantify visible floating anthropogenic macrodebris during hours of daylight (Figure 1, Tekman et al. in preparation).^32^ Each visual observation was conducted by a team of two scientists and lasted 1 h unless the ship reached a sampling station earlier. The maximum observation time per day depended on the total transit length and the prevailing physical conditions. When total transit lengths and physical conditions allowed, up to nine teams per day worked consecutively to maximize the area that was covered. One of the two observers located on the helicopter deck of the ship (15 m above sea level) identified floating items larger than 5 cm, and the other one noted the details. All anthropogenic items floating within an observation corridor (strip width) of 10 m starting behind the bow wave of the RV were recorded. A hand-held global positioning system device was used to record the position and time of the observations, and these waypoints were recorded for each item. The ship positions along the track of visual observations were downloaded from the ship’s position acquisition system (D-SHIP) in 1 min intervals, imported into ArcMap 10.6.1, and converted to continuous tracks, and the track lengths were measured using the “calculate geometry” function. The observation area of the transects was calculated by multiplying the track length by the strip width. The anthropogenic debris density of each observation transect was calculated by dividing the total number of items by the observed area, giving debris densities per transect (N km^–2^).

During the observations, large numbers of “white bits” smaller than 5 cm were observed. These items were classified separately because of their small size and the resulting uncertainty, even though their visual appearance (clear white color and sharp edges) indicated that they were not of a natural origin. The debris concentrations including and excluding the number of white bits were calculated separately due to the uncertainty in their material resulting from their small size. These concentrations were compared to the catamaran data. To set visual observations into context with the catamaran data, visual observations within a 250 km radius of the catamaran samples were grouped and mean values for the number of plastic items including and excluding white bits and number of white bits were calculated separately (Table S6). Statistical tests (Spearman’s rank correlation), calculation of GPS track lengths, and plots were carried out in RStudio v. 2022.12.0 + 353 using R (v. 4.2.2) and packages geosphere (v. 1.5–18), ggplot2 (v. 3.4.0), ggpubr (v. 0.5.0), scales (v. 1.2.1), and ggsci (v. 2.9).

## Results and Discussion

### QA/QC

In both process blank samples, no plastic items >330 μm were detected. Recovery experiments showed no loss of particles. Accordingly, no correction of the measured plastic item concentrations regarding systematic loss or blank contamination during sample treatment was required. Limits of detection (LODs) were calculated for a single detected plastic item and the respective sampled area and volume based on eqs 1 and 2. The LODs ranged from 558 to 924 items km^–2^ with a mean (±standard deviation, SD) of 720 ± 95 items km^–2^ for area-normalized concentrations and 0.0037 to 0.0062 items m^–3^ with a mean (±SD) of 0.0048 ± 0.0006 items m^–3^ for volume-based concentrations. No influence of the prevailing sea state on the efficiency of the net sampling was found (*R*^2^ = 0.241, *p* = 0.142).

### Distribution of Plastic Items

**Figure 2:**
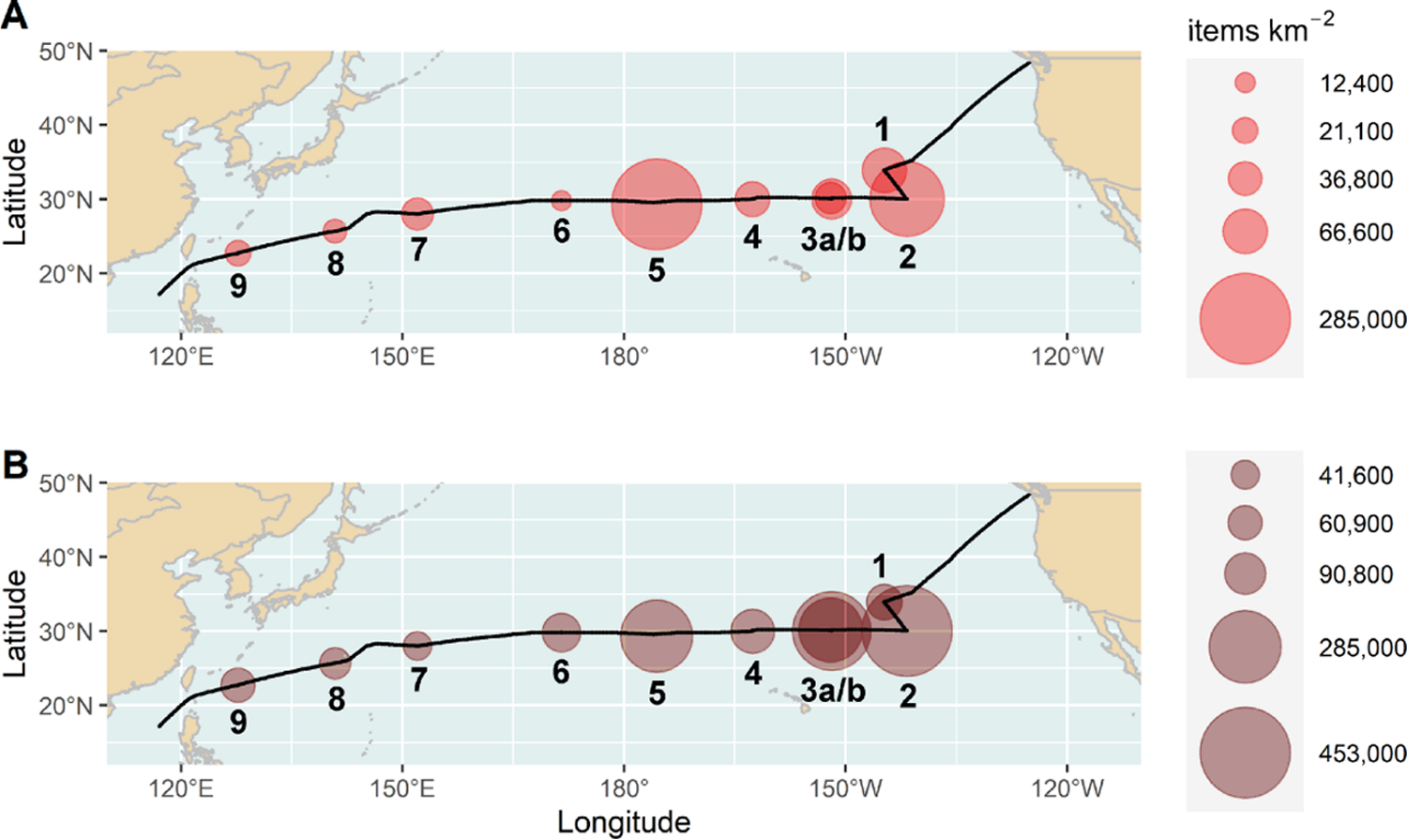
Distribution of (A) uncorrected concentrations and (B) wind-mixing-corrected concentrations integrated throughout the upper 5 m of the water body in items km^–2^ of surface-floating plastic items at nine sampling stations along the cruise track of RV SONNE on expedition SO268/3 from May to July 2019. The labels below the circles refer to station numbers. The size of the circles is proportional to the concentration of plastic items sampled in the neuston net (> 330 μm). The highest concentrations were recorded around the North Pacific Subtropical Gyre (stations 2, 3a/b) and the Papahanaumokuakea Marine National Monument (station 5).

A total of 1036 plastic items between 330 μm and 215 mm in size (Figure S4) were detected in North Pacific Ocean surface waters at nine stations along the cruise track and analyzed for size, polymer composition, shape, color, and visual presence of biofouling. Plastic items were present in all samples at (uncorrected) concentrations ranging from 12,400 to 285,200 items km^–2^ with a mean (±SD) of 75,200 ± 90,300 items km^–2^ (Figure 2A and Table S1). The highest (uncorrected) concentration of plastic items was recorded at the station within the Papaha̅naumokua̅kea Marine National Monument (PMNM, 285,200 items km^–2^; Figure 2A, station 5) followed by the NPSG (191,800 items km^–2^, Figure 2A, station 2). The lowest (uncorrected) plastic concentration was measured at the sampling site in the middle of the cruise track (12,400 items km^–2^; Figure 2A, station 6).

After correction for wind-induced vertical mixing, plastic item concentrations ranged from 41,600 to 452,800 items km^–2^ with a mean (±SD) of 170,300 ± 145,800 items km^–2^ (Figure 2B and Table S1). The highest corrected concentration was found at the stations in the NPSG (Figure 2B, stations 2, 3a/b, Table S1) followed by a similar level in the area of the PMNM (Figure 2B, station 5, Table S1). The lowest corrected concentration was recorded in the open ocean area southeast of Japan (Figure 2B, station 7, Table S1). The correction for wind-induced mixing led to higher plastic item concentrations at all sampling stations except stations 1 and 2, where the influence of wind-induced mixing was negligible because of a comparably calm sea (Beaufort sea states 1 and 2, respectively). The highest differences between measured and corrected concentrations were found at stations 3 and 6 with an increase of factors 6–7, where elevated wind speeds and a rougher sea (Beaufort sea state 5) led to stronger wind-induced mixing.

Duplicate sampling at station 3 showed uncorrected plastic item concentrations of 31,500 items km^–2^ and 52,800 items km^–2^ and depth-integrated concentrations of 223,900 items km^–2^ and 340,100 items km^–2^, resulting in variances of 40 and 34%, respectively. (Figure 2 and Table S1)

The quantities of plastic items found in the NPSG between the U.S. West Coast and Hawaii and for the North-West Pacific Ocean are in accordance with previous studies that showed similar concentrations ranging from 360 to 6,550,000 items km^–2^ (Table 1 and Figure S5). These data indicate that plastic items larger than 330 μm are omnipresent in surface waters of the North Pacific Ocean along the cruise track. Plastic item concentrations varied by a factor of 23 for measured uncorrected data and a factor of 10 for wind-mixing-corrected concentrations across the investigated area, which shows the heterogeneity of the plastic pollution and the differences between hotspots of plastic pollution and less contaminated areas. Duplicate sampling at station 3 indicated that concentrations of surface-floating plastic items varied even at small distances between sampling points. Besides the expected NPSG hotspot region between California and Hawaii that was confirmed by our data, we detected a second hotspot in the area of the PMNM (station 5) with comparably high concentrations, which was not predicted by the applied surface current model.^23^

**Table 1:** Abundance of Plastic Items Observed in Surface Water Studies in the Area between the U.S. West Coast and Hawaii (Figure S5)

study/lower size cutoff	location/time	concentration items km^–2^
this study/330 μm (uncorrected)	North Pacific Ocean/June 2019	72,200 (12,400–285,200)^a^
	NPSG/June 2019	42,100–191,800
	PMNM/June 2019	285,200
this study/330 μm (corrected)	North Pacific Ocean/June 2019	170,300 (41,600–452,800)^a^
	NPSG/June 2019	66,600–452,800
	PMNM/June 2019	285,200
Egger et al.^31^/500 μm (corrected)	outside NPSG/Aug. 2015 – Dec. 2019	16,468 (5,686–32,998)^b^
	Outer NPSG/Aug. 2015 – Dec. 2019	323,256 (57,578–470,330)^b^
	Inner NPSG/Aug. 2015 – Dec. 2019	773,114 (360,599–1,208,975)^b^
Pan et al.^33^/330 μm (uncorrected)	NW Pacific Ocean/Aug. – Sep. 2017	640–42,213
Goldstein et al.^34^/333 μm (uncorrected)	NPSG/Summer 2009	448,000 (7,000–3,211,000)^c^
	NPSG/Fall 2010	21,000 (2,000–682,000)^c^
Eriksen et al.^35^/335 μm (uncorrected)	NPSG/2007–2012	360–697,193

a Mean value and range in parentheses.

b Median values and 25–75th percentile of corrected concentrations using the same method as here.

c Median concentration converted from items m^–2^ to items km^–2^ (95% confidence intervals in parentheses).

The high concentration of plastic items detected in samples from the PMNM, a World Heritage Site, is remarkable and was not predicted by the SCUD model. There may be other factors than large-scale circulations that could explain the high concentration of plastic items at this site: (A) in an earlier study covering the beaches of the Northwestern and main Hawaiian Islands, the highest plastic particle concentrations were found in sediments of the Midway Atoll.^36^ Particles from nearby beaches could have been remobilized by winds and waves, explaining the high concentration of plastic debris in surface waters recorded in near open ocean areas.^37,38^ (B) Furthermore, the formation of surface windrows could lead to the accumulation of surface-floating particles, explaining the co-occurring high amount of biogenic material in the sample from this area (Figure S6).^39^ Since the determined distribution pattern is based on single samples (except station 3), it must be viewed with caution. To further support the data, the sample-based distribution pattern was compared to the distribution of larger plastic items obtained from the visual observations.

### Comparison of Small Plastic Items and Macroplastic Concentrations

A total of 6863 debris items were recorded during 152 visual observations covering 34.63 km^2^. Plastic accounted for 99% (6812 items) of the total debris count. In addition, 15,355 white bits were noted from 142 visual observations. For the calculation of plastic concentrations including the white bits, 10 transects had to be excluded from the analyses because of missing data. The mean plastic debris concentration along the cruise track was 197 items km^–2^ or 677 items km^–2^ when the white bits were included in analyses (Tekman et al., in preparation). Spearman’s rank correlation (Figure S7) showed a strong and significant positive correlation between the plastic item concentration from catamaran samples and from visual observations excluding (*R*_s_ = 0.88, *p* < 0.05, *n* = 9) and including white bits (*R*_s_ = 0.87, *p* < 0.05, *n* = 9).

**Figure 3:**
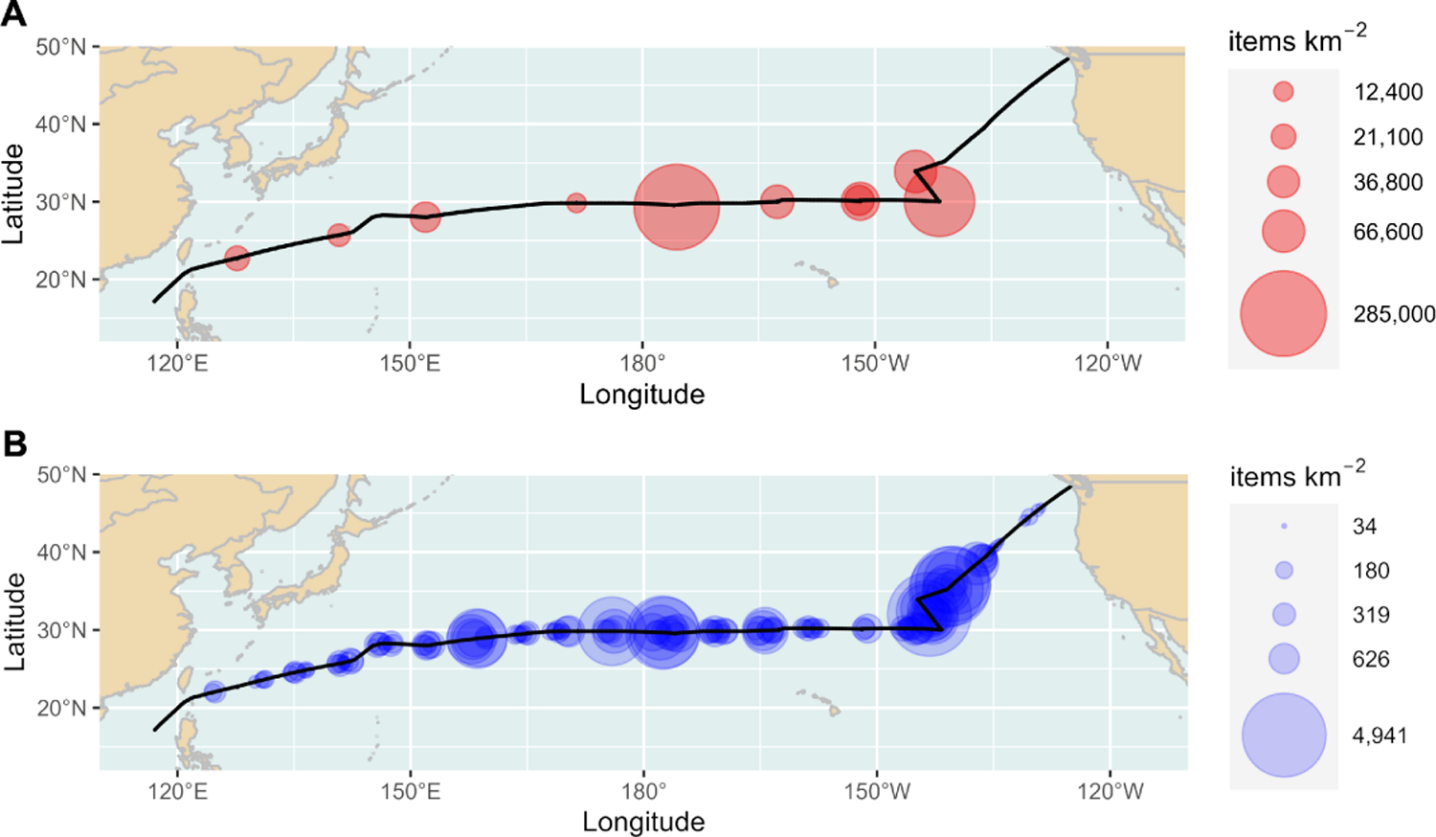
Comparison of uncorrected plastic debris concentrations in items km^–2^ recorded from catamaran samples (A, red) and visual observations (B, blue). Correlation of data from A and B: *R* = 0.87, *p* < 0.05. Please refer to Figure S7 for details.

A positive correlation was also found between the white bits only and the total concentrations of plastic items >330 μm in catamaran samples (*R*_s_ = 0.73, *p* < 0.05, *n* = 9). Occasionally, plastic particles that may have been perceived as “white bits” were detected in the neuston samples collected along the cruise track (Figure S4). Furthermore, no nonplastic particles meeting the color and size criteria of the “white bits” were found. This supports the interpretation that these white bits are likely plastic items. Furthermore, the correlation between concentrations of plastic items found in the neuston samples and the observed “white bits” supports this interpretation (Figures 3 and S7). However, the number of these fragments found in the net was low and did not allow for a direct correlation with the numbers detected by visual observations. This may be explained by the fact that visual observations cover a much wider area (with a strip width of 10 m) compared with the net (opening width of 0.3 m).

The strong correlation between the plastic items from catamaran samples and the observed large plastic debris suggests that visual observations can be used as indicators for possible accumulation areas of microplastic particles in surface waters (Figure 3). This is supported by previous studies, e.g., at basin scale in the Black Sea area, finding increasing proportions of even smaller particles down to a size of 2.5 cm.^40^ Furthermore, a study conducted in the Northeast Pacific Ocean showed a positive correlation between plastic items detected by net tows and visual observations at larger spatial scales.^34^ Therefore, the detection of areas with elevated concentrations of floating macroplastic could help to identify hotspots of microplastic pollution.

The visual surveys can be conducted opportunistically, as they do not require dedicated ship time. Certainly, physical factors (e.g., sea state, light, observer experience) introduce some uncertainty compared to sampling methods, but the experienced observers provided detailed instructions and regular quality control of the teams to ensure comparability of data and such a large area as our study could only be surveyed for floating debris with an observational program.^41^

### Size Groups of Plastic Items from Catamaran Samples

**Figure 4:**
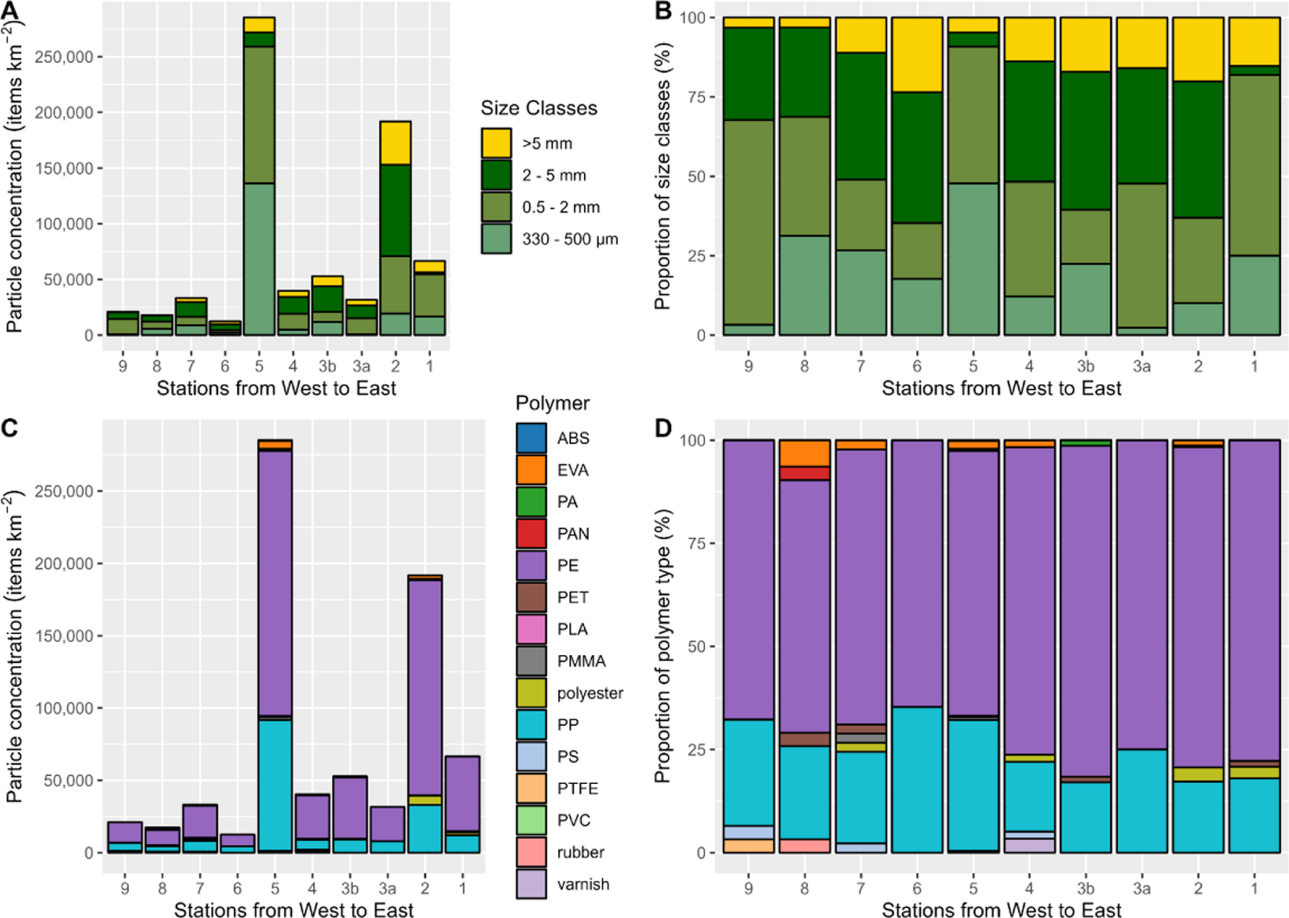
Characteristics of plastic items from all sampling stations along the cruise track. (A) Absolute concentrations of different size classes (items km^–2^). (B) Proportions of items from different size classes [%]. (C) Concentrations of different polymer classes (items km^–2^). (D) Proportions of items of different polymer types (%) (ABS, acrylonitrile butadiene styrene; EVA, ethylene-vinyl acetate; PA, polyamide; PAN, polyacrylonitrile; PE, polyethylene; PET, poly(ethylene terephthalate); PLA, polylactic acid; PMMA, poly(methyl methacrylate); PP, polypropylene; PS, polystyrene; PTFE, poly(tetrafluoroethylene); and PVC, poly(vinyl chloride)).

Most of the recorded plastic items (89%) were smaller than 5 mm and were thus categorized as microplastics.^42^ Microplastics in the size range of 330–500 μm accounted for 29% of all items sampled along the cruise track (Figures 4A,B and S8). One of the most interesting findings of this study is that 69% of these plastic items at the lower size limit of the used method were obtained from the one sample gathered in the PMNM region, highlighting the pollution by small microplastic particles in this area. This observation could indicate a higher degree of weathering-related fragmentation or different accumulation mechanisms.^43,44^ In contrast, the highly contaminated sample from the NPSG area (station 2) was dominated by items between 2 and 5 mm (42%) and higher shares of macroplastic debris (20%) than the sample from the PMNM (5%). This distribution further supports the existence of different accumulation mechanisms for both hotspot regions. It should be noted that the high contribution of the smallest size fraction at the PMNM observed in this study suggests the potential presence of more particles in yet lower size ranges below the size cutoff of our sampling method, similar to previous studies.^22,45,46^ This small micro- to nanoplastic fraction should be under future investigation once adequate analytical tools are available.

### Polymer Types

Polyethylene (PE, 71%) and polypropylene (PP, 24%) were the most abundant polymers (Figure 4C,D) concurring with observations from previous studies from the North Pacific^22,33,47^ and other regions of the World’s Oceans.^48,49^ Having polymer densities lower than seawater (0.87–0.97 and 0.90–0.92 g/cm^3^, respectively), these polymers are expected to be present at the ocean surface. Furthermore, both polymers have a combined market share of 40–50% and are used in a wide range of (single-use) applications.^50^ Consequently, high amounts of PE and PP are expected at the sea surface, which is consistent with the findings of the current study. Ethylene-vinyl acetate (EVA), which is frequently used in sports and fishing applications, accounted for 1.4%, and polyesters, which are widely used in the textile industry and maritime shipping, accounted for 1.2% of all plastic items detected. Low numbers of particles of polystyrene (PS), poly(methyl methacrylate) (PMMA), polyamide (PA), poly(ethylene terephthalate) (PET) (0.4% each), PTFE, PVC, polyacrylonitrile (PAN), polylactic acid (PLA), acrylonitrile butadiene styrene (ABS), and rubber (0.1% each) were detected only occasionally, concurring with the fact that they are unlikely to float on the ocean surface given their specific densities. However, due to wind-induced vertical mixing^30^ or sea surface microlayer tension,^51^ it is not surprising to find these particles floating at the ocean surface.^45^ Detected varnish particles (0.2%) could be ship paint from the RV or other ships.^52^

### Shape, Color, and Biofouling

**Figure 5:**
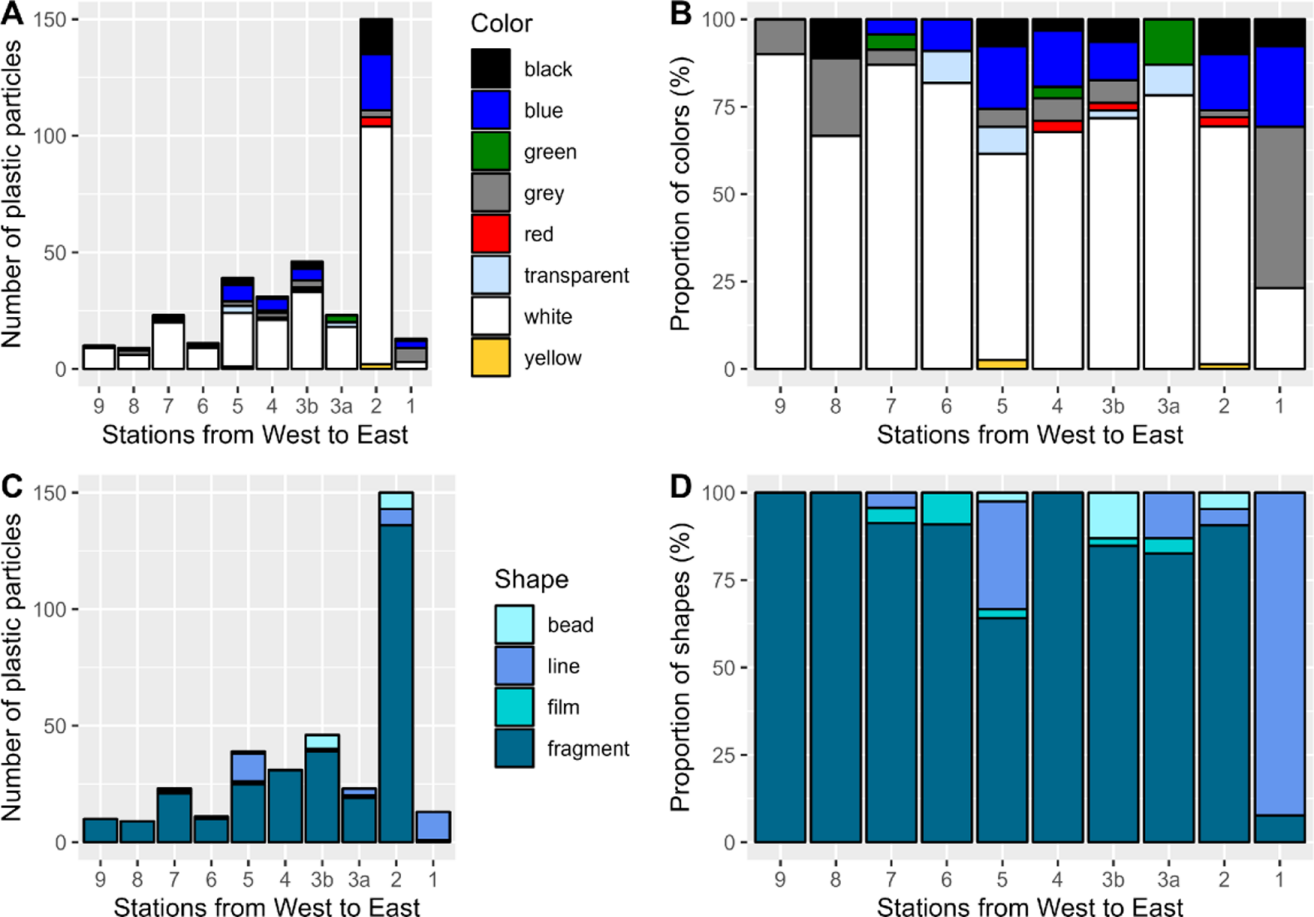
Visual characteristics of plastic items larger than 2 mm. (A) Absolute numbers of plastic items of different colors. (B) Proportions of different colors. (C) Absolute numbers of plastic items of different shapes. (D) Proportion of different shapes.

A total of 355 plastic items larger than 2 mm were further characterized in terms of shape, color, and signs of biofouling. The most abundant colors were white (69%), blue (13%), black (6.8%), and gray (5.6%). Transparent, red, green, and yellow particles accounted for less than 2% each (Figure 5A,B). The classification of color was, in many cases, complicated by weathering-induced discoloration, leading to a strong similarity between pale-colored and white particles. The high share of pale-colored and white particles can be interpreted as a visual sign of advanced weathering by sunlight and has also been observed in previous studies.^53−58^ Furthermore, 38% of plastic particles were visually characterized as biofouled. Previous studies have shown that more white or pale-colored particles are ingested by seabirds, possibly because of their general high abundance in surface waters.^59−61^ Moreover, biofouled plastics could have a stronger and more natural chemical signature, making them more attractive as food to selective feeders among zooplankton, fish, birds, and turtles.^62−65^ Additionally, a noticeable amount of brown, jellylike biogenic material was found in the sample from the PMNM area (Figure S6).

The majority of the detected plastic items larger than 2 mm consisted of fragments (85%), followed by lines and filaments (9.9%), beads (3.9%), and films (1.4%) (Figure 5C,D). No foam-like items (e.g., expanded PS or EVA foams) were detected. The high share of fragments can also be interpreted as a sign of advanced weathering and fragmentation due to environmental factors such as UV radiation, biological processes, and mechanical aging, e.g., from friction, abrasion, or wave movements in water, in combination with long-term exposure to these factors due to long residence times.^58,66,67^ The sample from station 1 clearly differed in the proportions of particle shapes, showing a high share of lines and filaments (92%) and a low share of fragments (8%) in the size fraction larger than 2 mm, whereas all other samples were dominated by fragments (≥64%, Figure 5C,D). This, in combination with these lines and filaments being made of PE (75%), polyester (13%), PP, and PA (6% each), indicates that the plastic pollution at this sampling site could have been caused by fishing activities, which is consistent with a previous study conducted in the area of the NPSG that detected high amounts of debris from the fishing industry.^12^ The dissimilarity of particle shapes found at stations next to each other (520 km distance), in addition to the large difference in concentrations (66,600 N km^–2^ for station 1 and 191,800 N km^–2^ for station 2) in the NPSG, underlines the high heterogeneity of plastic pollution in this accumulation zone. Smaller plastic particles less than 2 mm in size were not extensively examined for their color and shape but they showed a similar distribution consisting mostly of fragments and white or pale particles (Figure S9).

### Implications

Our results support that floating plastic debris (>330 μm) is ubiquitous between 20°N and 30°N across the North Pacific Ocean as predicted by surface current modeling.^23^ Besides the predicted and well-known accumulation zone in the area of the NPSG, our data indicate an unpredicted hotspot of smaller plastic items in the area of the PMNM with a comparable number concentration of plastic items but a higher share of smaller particles in the range of 330–500 μm. These findings imply that the used surface current model parameterizations and setup cannot fully forecast the densities of floating plastic since mesoscale and sub-mesoscale processes may also be relevant.^23^ So far, studies have shown that plastic macrodebris accumulates in the Subtropical Convergence Zone where the PMNM is located, but there are only limited data available on floating microplastic particles in this area.^37,38^ The detected distribution pattern is also supported by the visual observations, which showed large amounts of rather small debris (“white bits”) in this region. Furthermore, the co-occurrence of a noticeable amount of biogenic material in the sample from the PMNM region (Figure S6) indicates that heteroaggregation could be a contributing factor, which is supported by recent data suggesting that the abundance of neuston organisms is highest in areas of high plastic concentrations due to similar transport mechanisms.^68^ This observation may result from meso- and sub-mesoscale accumulation mechanisms, such as surface windrows and slicks.^39,69^

The high share of plastic items in the size range of 330–500 μm in the sample from the PMNM (Figure S8), one of the largest marine conservation areas in the world, where other environmental stressors already threaten biodiversity, highlights potential ecological repercussions. These rather small plastic items are more likely to be ingested by a wider range of species and tend to more rapidly sorb or desorb chemical additives, pollutants, and pathogens because of their higher surface-to-volume ratio.^17^ Furthermore, about 28% of plastic items >2 mm were classified as biofouled, making these particles potentially more attractive as food and increasing the chance of consumption by marine biota.^62−65^ The combination of high concentrations, small size, and higher likelihood for ingestion poses a potential threat to various marine species like Laysan albatrosses and other seabirds, which use the Subtropical Convergence Zone as the foraging ground.^70,71^ Additionally, a study performed in coastal waters of Hawaii showed that the density of larval fish and plastic debris is higher in areas with increased planktonic content, threatening these fish in a critical stage of life.^72^ The combination of a noticeable amount of biogenic material and especially small plastic items in the sample from the PMNM region indicates a potential threat to larval fish feeding inside this accumulation zone.

Overall, our data support the widespread distribution and elevated concentrations of plastic items in the middle of the ocean, far from human activities, highlighting the need to address plastic pollution efficiently at a global level.^73^ The combination of a high share of fragments, the increasing concentrations of plastic items with decreasing particle size, the high amount of white or pale-colored items, and the strong positive correlation between small and large plastic items could also be seen as an indication of ongoing fragmentation processes in the marine environment, induced by weathering processes. Our results indicate that visual observations could be a useful tool to provide an overview of the large-scale pollution of the global ocean surface waters by using the concentration of observed (macro)plastic debris as a proxy for microsized plastic items. The correlation between visually observable plastic debris and surface-floating (micro)plastic items should be further investigated to improve our knowledge of the large-scale distribution of plastic pollution without the need for using sophisticated, labor-intensive, and time-consuming methods for sampling, extraction, and quantification of (micro)plastic particles.

## Supporting Information Available

Additional text; sample data and environmental parameters for all catamaran trawls; classification of plastic items in different sizes and type classes; concentrations of plastic items from catamaran samples and visual surveys; sampling gear used for the sampling of the surface-floating plastic item; visual comparison of samples from PMNM (left) and NPSG areas (right) before digestion; and size distribution of all detected plastic items in catamaran samples along the cruise track (PDF)
