# Supplementary material for: Hotspots of Floating Plastic Particles across the North Pacific Ocean
Source: Environ Sci Technol. 2024 Feb 23;58(9):4302–13. doi: 10.1021/acs.est.3c05039 (PMC10919090; doi:10.1021/acs.est.3c05039)
Supplement: Supplementary file 1 — es3c05039_si_001.pdf [file es3c05039_si_001.pdf]

Supporting information for "Hotspots of Floating Plastic Particles across the North Pacific Ocean"

Robby Rynek[a,I], Mine B. Tekman[b,c] Christoph Rummel[d,II], Melanie Bergmann[b], Stephan Wagner[a,III], Annika Jahnke[e,f], Thorsten Reemtsma*[a,g]

[a] Department of Analytical Chemistry, Helmholtz Centre for Environmental Research – UFZ, 04318 Leipzig, Germany

[b] Alfred-Wegener-Institut, Helmholtz-Zentrum für Polar- und Meeresforschung, 27570 Bremerhaven, Germany

[c] Department of Natural and Mathematical Sciences, Faculty of Engineering, Ozyegin University , Istanbul. Turkey

[d] Department of Bioanalytical Ecotoxicology, Helmholtz-Centre for Environmental Research – UFZ, 04318 Leipzig, Germany

[e] Department of Exposure Science, Helmholtz-Centre for Environmental Research – UFZ, 04318 Leipzig, Germany

[f] Institute for Environmental Research, RWTH Aachen University, 52047 Aachen, Germany

[g] Institute of Analytical Chemistry, University of Leipzig, Linnéstrasse 3, 04103 Leipzig, Germany

*) corresponding author

Number of pages: 13

2 texts, 9 figures and 6 tables

Present addresses:
[I] Department of Monitoring and Exploration Technologies, Helmholtz Centre for Environmental Research – UFZ, 04318 Leipzig, Germany
[II] German Environment Agency, Section II 2.3 "Protection of the Seas and Polar Regions", 06844 Dessau-Roßlau, Germany
[III] Institute for Analytical Research, Hochschule Fresenius GmbH, 65510 Idstein, Germany

Table S1. Sample data and environmental parameters for all catamaran trawls. Longitude and latitude represent the start of the tow.

Station	Start of sampling (UTC)	Longitude	Latitude	Towing distance (flowmeter) [m]	Towing distance (GPS) [m]	Water volume [m³]	Plastic item conc. [items km-2]	Plastic item conc. [items m-3]	Wind speed [m/s]	Beaufort sea state	Corrected plastic item conc. [items km-2]
**1**	2019-06-05, 06:28:00	144.75958 °W	33.89911 °N	3,604.2	4284.9	162.2	66,600	0.222	0.94	1	66,600
**2**	2019-06-07, 01:43:00	141.68896 °W	30.05341 °N	4,136.7	4355.4	186.2	191,800	0.639	7.48	4	452,800
**3a**	2019-06-08, 22:28:00	151.90303 °W	30.05910 °N	4,805.1	6289.0	216.2	31,500	0.105	9.13	5	223,900
**3b**	2019-06-09, 17:10:00	152.02939 °W	30.16941 °N	4,651.5	5526.8	209.3	52,700	0.176	9.45	5	340,100
**4**	2019-06-12, 01:41:00	162.60483 °W	29.99351 °N	4,874.4	6434.2	219.3	40,300	0.135	7.12	4	102,700
**5**	2019-06-15, 05:32:00	175.58116 °W	29.31139 °N	4,943.4	5836.0	222.5	285,200	0.951	1.73	2	285,200
**6**	2019-06-18, 04:20:00	171.50209 °E	29.83496 °N	4,577.7	6157.3	206.0	12,400	0.042	9.57	5	78,900
**7**	2019-06-22, 05:21:00	152.01876 °E	28.02121 °N	4,522.2	6157.3	203.5	33,200	0.111	4.79	3	41,600
**8**	2019-06-24, 06:20:00	140.87607 °E	25.68980 °N	5,971.5	6704.5	268.7	17,300	0.058	6.14	4	50,700
**9**	2019-06-27, 04:54:00	127.72646 °E	22.70671 °N	4,897.2	6603.8	220.4	21,100	0.071	6.63	4	60,900

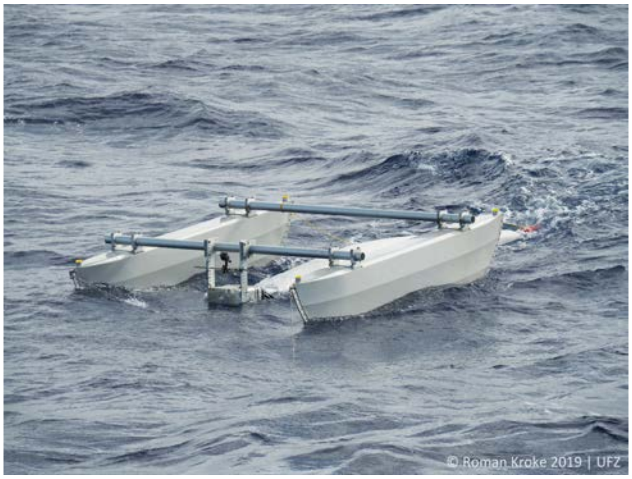

Figure S1. Sampling gear used for the sampling of surface floating plastic items. The device consists of a neuston net with a mesh size of 330 µm attached to a catamaran. (Copyright: Roman Kroke / UFZ)

Text S1. Enzymatic purification protocol. A basic enzymatic purification protocol was used to remove natural particles from the samples.[1] Samples were rinsed into stainless steel reactors semi-enclosed with 10 µm stainless steel filters. Subsequently, the samples were treated with sodium dodecyl sulfate (*SDS*, J.T.Baker, USA), Protease A-01, Cellulase TXL, Chitinase (all ASA Spezialenzyme GmbH, Germany) and hydrogen peroxide (H_2O_2, CHEMSOLUTE, Germany) in an Unimax 1010 incubation shaker (Heidolph Instruments GmbH & Co. KG, Germany). Enzyme reaction solutions were prepared by dissolving the enzyme solution in phosphate-buffered saline (PBS), prepared by dissolving 8.00 g sodium chloride (NaCl, AppliChem GmbH, Germany), 0.20 g potassium chloride (KCl), 1.42 g disodium hydrogen phophate (Na_2HPO_4) and 0.27 g potassium dihydrogen phosphate (KH_2PO_4) (Merck KGaA, Germany) in 1 L of Milli-Q water. The optimum pH for each enzyme was adjusted by adding diluted sodium hydroxide (NaOH, Merck KGaA, Germany) or hydrochloric acid (HCl, CHEMSOLUTE, Germany).

The samples were treated according to the following scheme:

1. 60 mL SDS, 5% (w/w) in Milli-Q water, 1 d, 50 °C
2. 5 mL Protease A-01 in 25 mL PBS, 1 d, 50°C, pH 9
3. 10 mL Cellulase TXL in 50 mL PBS, 4 d, 50°C, pH 5
4. 50 mL H_2O_2 (35%), 1 d, 50°C
5. 2 mL Chitinase in 30 mL PBS, 5 d, 37°C, pH 5
6. 50 mL H_2O_2 (35%), 1 d, 37°C

After each step, the content of the reactors was filtered through the 10 µm stainless steel filter at their ends and the reactors were rinsed with 1 L Milli-Q water to remove the reagents.

Text S2. Calculations of plastic item concentrations. All area-based concentrations were calculated using flowmeter data. To evaluate the performance of the flowmeter during different sampling conditions, the deviation between calculated areas based on flowmeter and GPS track data (Table S1) was compared for each Beaufort sea state. (Figure S1)

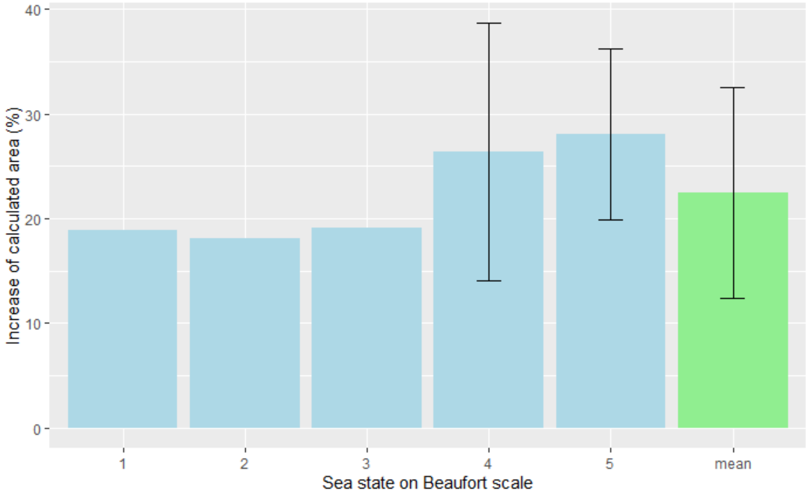

Figure S2. Increase of calculated sampling area based on GPS track length compared to flowmeter data at different sea states (blue) and mean value (green).

Deviations of calculated concentrations ranged from 5.3% to 34.8% with a mean of 22.5% ± 10.1% with no clear relationship to the prevailing sea state. Although the concentrations derived from GPS data were lower than those determined from flowmeter measurements, the pattern of distribution remained consistent. Thus, concentrations derived from flowmeter data were used throughout the manuscript.

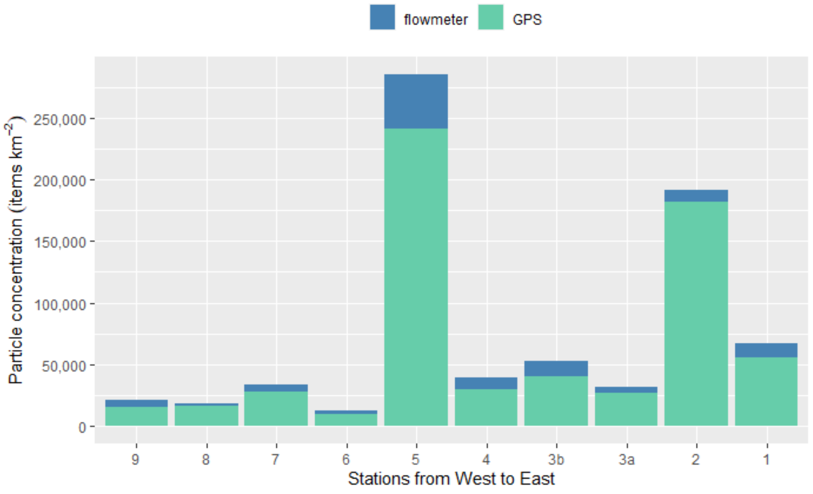

Figure S3. Comparison of plastic particle concentrations in items km^{-2} based on flowmeter data (blue) and GPS tracks (green) for all sampling stations.

Buoyant plastic particles can be vertically distributed in the upper layers of the water column due to wind-induced vertical mixing. Therefore, particle concentrations determined by surface water sampling techniques like catamaran trawling are dependent on the prevailing sea states. To level out the influence of different sea states during sampling and improve the comparability of results between stations and towards published data sets, measured plastic concentrations were corrected for wind-induced vertical mixing using on a 1-D model developed by Kukulka et al. 2012.[2] Corrected concentrations were calculated by means of Eq. S1.

$$c_i = \frac{c_A}{F} = \frac{c_A}{1-e^{-dw_b A_0^{-1}}} \quad \text{(S1)}$$

The measured area-based particle concentration c_A in items km^{-2} is corrected for vertical mixing caused by the prevailing sea state, taking into account the sampling depth d, the median terminal rising velocity w_b (m s^{-1}) and the near-surface turbulent exchange coefficient A_0. Additional information on these calculations can be found in Lebreton et al. 2018.[3] Since the terminal rising velocity w_b depends on the type and size of a particle, all detected plastic items were classified regarding size and type (Table S2). Plastic items in the size range of 330-500 µm were grouped together with the size range of 0.05-0.15 cm, assuming a similar behavior during wind-induced vertical mixing.

Table S2. Classification of plastic items in different size and type classes according to Lebreton et al.[3]

	**<0.15 cm**	**0.15-0.5 cm**	**0.5-1.5 cm**	**1.5-5 cm**	**5-10 cm**	**10-50 cm**
**fragments, hard plastic items, sheets and films**	H1	H2	H3	H4	H5	H6
**plastic lines, ropes and nets**	N1	N2	N3	N4	N5	N6
**pre-production plastic pellets**	P1	P2	-	-	-	-

Area-based concentrations c_A of plastic items determined at each station were calculated for every type and size class (Table S3). Median correction factors F for every type and size class were extracted from Lebreton et al. (Table S4) and used to calculate depth-integrated concentration c_i based on Eq. 3 (Table S5). [3]

Table S3. Concentration in items km^{-2} of plastic items larger than 330 µm according to the classes specified in Table S2 in the catamaran samples for the stations 1 to 9 (details given in Table S1).

station / class	1	2	3a	3b	4	5	6	7	8	9
**H1**	54,566	70,910	15,049	20,811	19,148	258,931	4,369	16,216	12,281	14,294
**H2**	925	76,551	11,466	18,730	15,045	11,463	5,097	13,268	4,466	6,126
**H3**	0	31,426	2,150	8,324	6,155	5,394	2,913	2,948	558	681
**H4**	0	1,612	717	694	0	0	0	0	0	0
**H5**	0	0	0	0	0	674	0	0	0	0
**H6**	0	0	0	0	0	0	0	0	0	0
**N1**	0	0	0	0	0	0	0	0	0	0
**N2**	925	0	0	0	0	0	0	0	0	0
**N3**	5,549	3,223	0	0	0	5,394	0	0	0	0
**N4**	2,775	2,417	1,433	0	0	2,697	0	737	0	0
**N5**	0	0	0	0	0	0	0	0	0	0
**N6**	1,850	0	717	0	0	0	0	0	0	0
**P1**	0	0	0	0	0	0	0	0	0	0
**P2**	0	5,641	0	4,162	0	674	0	0	0	0
**total**	**66,589**	**191,779**	**31,531**	**52,722**	**40,347**	**285,229**	**12,379**	**33,170**	**17,304**	**21,100**

Table S4. Median correction factors F for correction of the concentrations of plastic items for wind-induced vertical mixing for different particle classes specified in Table S2 based on prevailing sea states taken from Lebreton et al. 2018.[3]

station / class	1	2	3a	3b	4	5	6	7	8	9
**H1**	1	0.3	0.1	0.1	0.3	1	0.1	0.7	0.3	0.3
**H2**	1	0.5	0.2	0.2	0.5	1	0.2	0.9	0.5	0.5
**H3**	1	0.7	0.3	0.3	0.7	1	0.3	1	0.7	0.7
**H4**	1	0.7	0.3	0.3	0.7	1	0.3	1	0.7	0.7
**H5**	1	0.8	0.4	0.4	0.8	1	0.4	1	0.8	0.8
**H6**	1	0.9	0.5	0.5	0.9	1	0.5	1	0.9	0.9
**N1**	1	0.1	0.1	0.1	0.1	0.9	0.1	0.3	0.1	0.1
**N2**	1	0.2	0.1	0.1	0.2	1	0.1	0.4	0.2	0.2
**N3**	1	0.5	0.2	0.2	0.5	1	0.2	0.9	0.5	0.5
**N4**	1	0.7	0.3	0.3	0.7	1	0.3	1	0.7	0.7
**N5**	1	0.6	0.2	0.2	0.6	1	0.2	0.9	0.6	0.6
**N6**	1	0.8	0.4	0.4	0.8	1	0.4	1	0.8	0.8
**P1**	1	0.7	0.3	0.3	0.7	1	0.3	1	0.7	0.7
**P2**	1	0.9	0.5	0.5	0.9	1	0.5	1	0.9	0.9

Table S5. Depth-integrated concentrations in items km^{-2} of plastic items after correction for wind-induced vertical mixing according to the classes specified in Table S2 in the catamaran samples for the stations 1 to 9.

class \ station	1	2	3a	3b	4	5	6	7	8	9
**H1**	54,566	236,367	150,489	208,112	63,826	258,931	43,690	23,166	40,935	47,646
**H2**	925	153,101	57,329	93,650	30,089	11,463	25,486	14,742	8,931	12,252
**H3**	0	44,894	7,166	27,748	8,792	5,394	9,709	2,948	797	972
**H4**	0	2,302	2,389	2,312	0	0	0	0	0	0
**H5**	0	0	0	0	0	674	0	0	0	0
**H6**	0	0	0	0	0	0	0	0	0	0
**N1**	0	0	0	0	0	0	0	0	0	0
**N2**	925	0	0	0	0	0	0	0	0	0
**N3**	5,549	6,446	0	0	0	5,394	0	0	0	0
**N4**	2,775	3,453	4,777	0	0	2,697	0	737	0	0
**N5**	0	0	0	0	0	0	0	0	0	0
**N6**	1,850	0	1,792	0	0	0	0	0	0	0
**P1**	0	0	0	0	0	0	0	0	0	0
**P2**	0	6,267	0	8,324	0	674	0	0	0	0
**total**	**66,589**	**452,831**	**223,942**	**340,148**	**102,707**	**285,229**	**78,885**	**41,594**	**50,664**	**60,871**

Table S6. Concentrations of plastic items from catamaran samples and visual surveys.

station / group	measured plastic item concentration [items km^{-2}]	visual survey plastic items concentration [items km^{-2}]	visual survey white bit concentration [items km^{-2}]	visual survey total concentration [items km^{-2}]
**1**	66,600	535	1,179	1,713
**2**	191,800	313	786	1,100
**3**	42,100	211	154	365
**4**	40,300	205	434	639
**5**	285,200	242	1,329	1,571
**6**	12,400	96	208	303
**7**	33,200	172	171	343
**8**	17,300	110	130	240
**9**	21,100	30	52	82

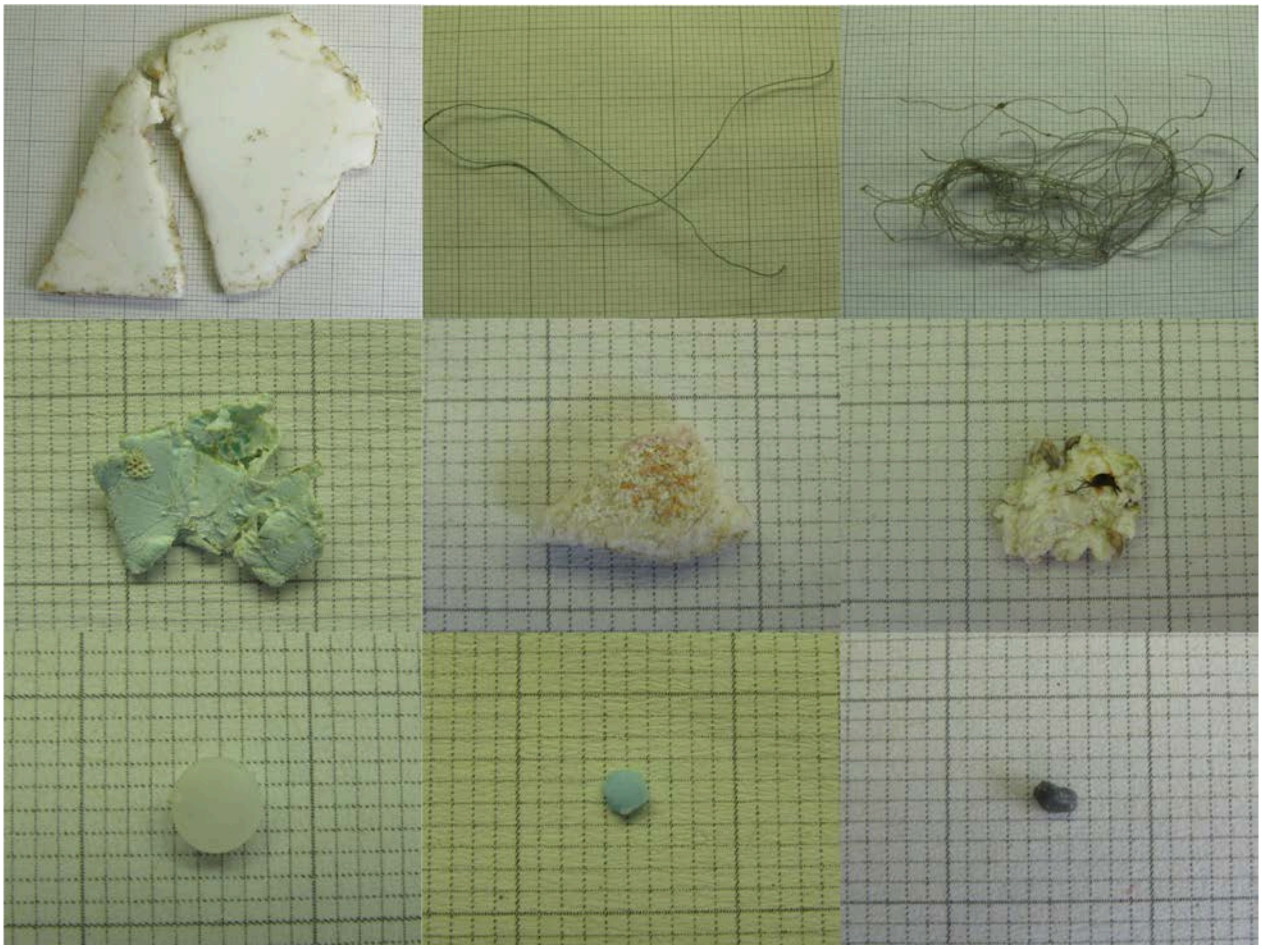

Figure S4. Images of selected plastic items of different sizes, shapes, colors and weathering states found in catamaran samples along the cruise track. Images were taken on graph paper with a size of 1x1 mm for each small square to ensure size comparability.

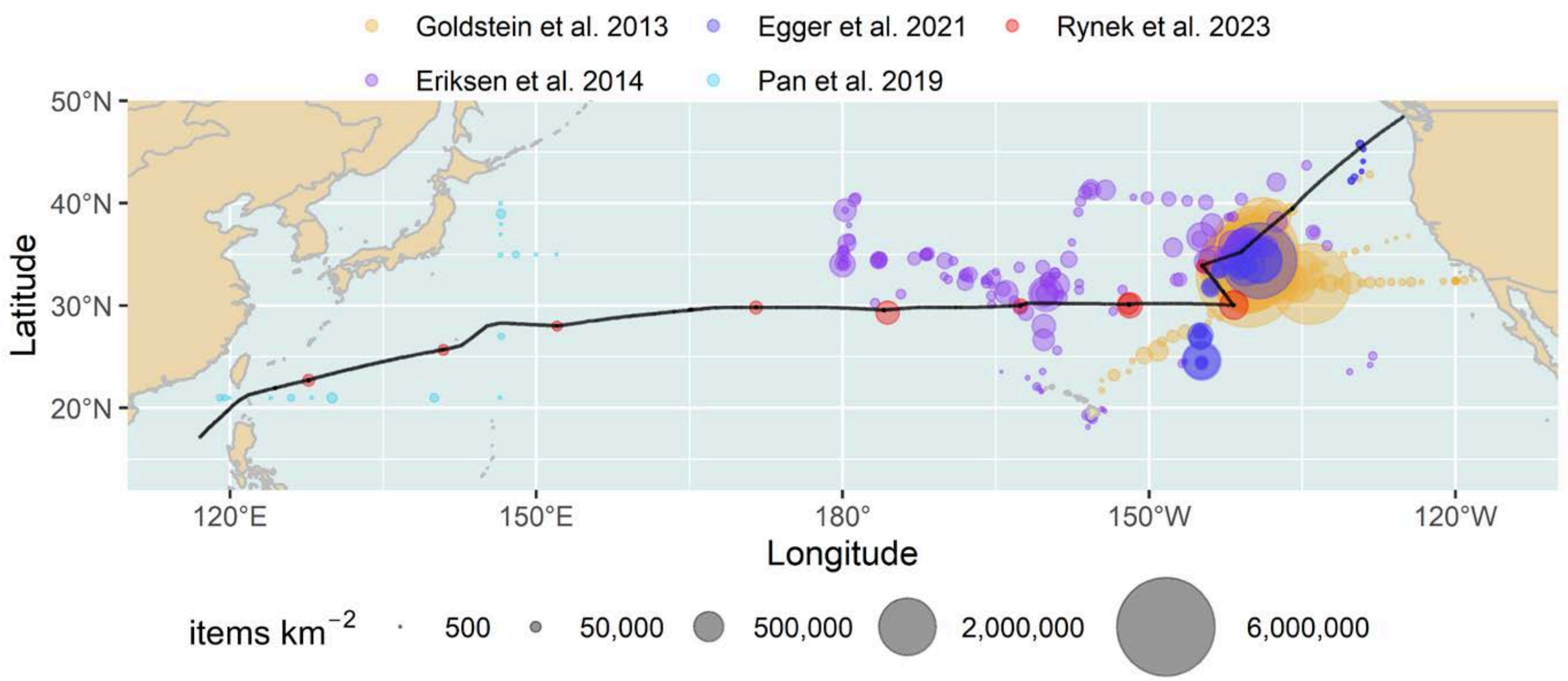

Figure S5. Reported concentrations of surface-floating (micro)plastic particles across the North Pacific Ocean in items km^{-2}. The area of the circles is proportional to the concentration.[4–7]

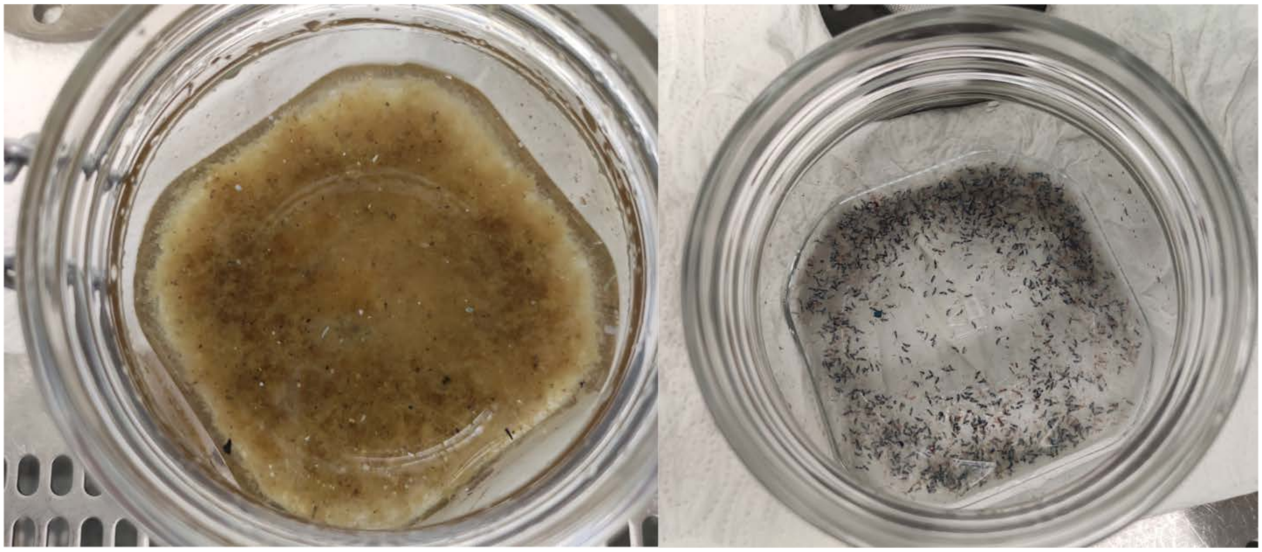

Figure S6. Visual comparison of samples from PMNM (left) and NPSG area (right) before digestion. The sample from the PMNM area had a noticeable presence of organic material and a jelly-like consistency, whereas all other samples showed a substantially lower content of suspended matter.

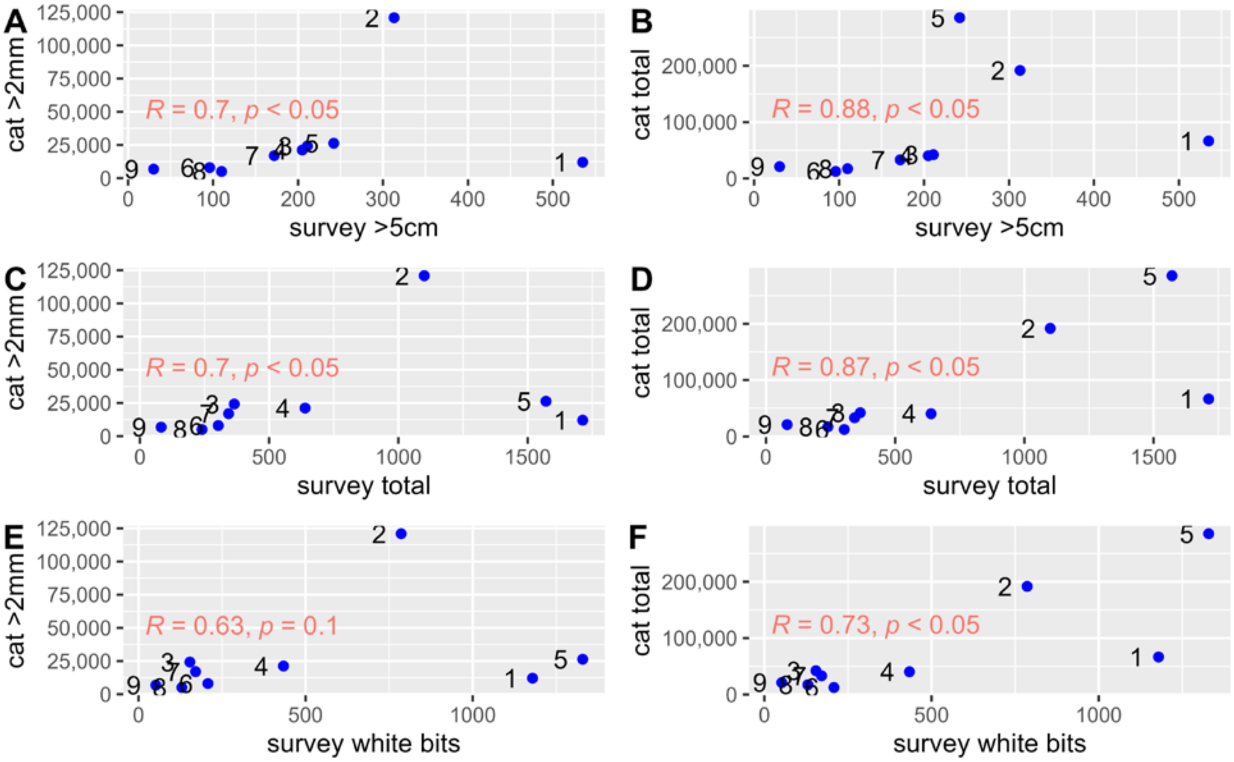

Figure S7. Correlation plots between concentrations (items km^{-2}) of plastic items in catamaran samples and visual surveys in 250 km proximity with correlation measure R and significance p of the Spearman rank correlation. A – Plastic items >2 mm from catamaran samples and visually observed plastic debris excluding white bits. B – Total plastic items from catamaran samples and visually observed plastic debris excluding white bits. C - Plastic items >2 mm from catamaran samples and visually observed plastic debris including white bits. D - Total plastic items from catamaran samples and visually observed plastic debris including white bits. E - Plastic items >2 mm from catamaran samples and visually observed white bits. F - Total plastic items from catamaran samples and visually observed white bits.

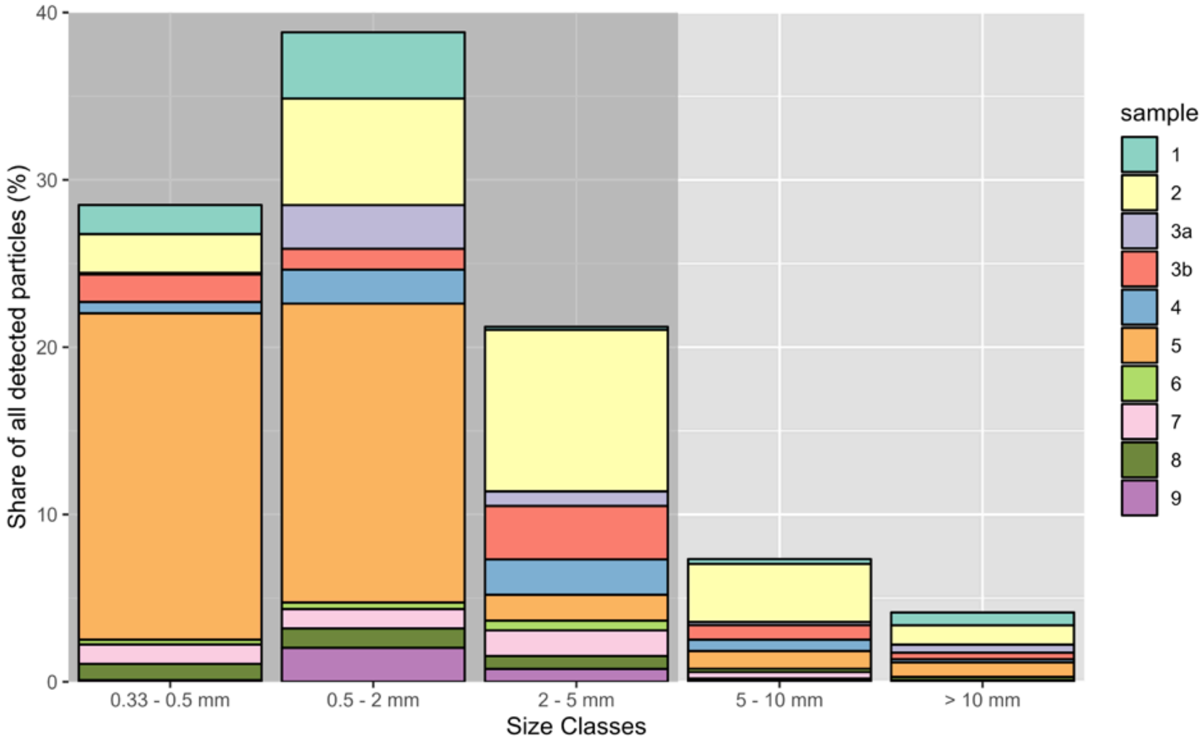

Figure S8. Size distribution of all detected plastic items in catamaran samples along the cruise track. Bars with dark grey background represent microplastic items (< 5 mm), bars with light grey background represent macroplastic items (> 5 mm).

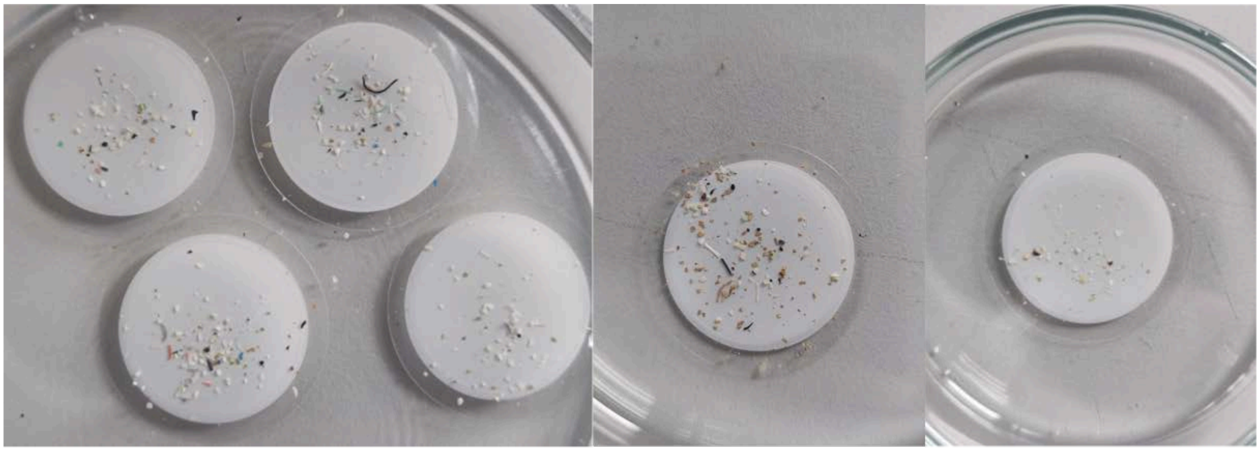

Figure S9. Photographs of items from the 300-500 μm size fraction deployed on Anodisc filters after enzymatic digestion. Plastic items are dominated by fragments and are mostly white or pale-colored.

References

(1) Löder, M. G. J.; Imhof, H. K.; Ladehoff, M.; Löschel, L. A.; Lorenz, C.; Mintenig, S.; Piehl, S.; Primpke, S.; Schrank, I.; Laforsch, C.; Gerdts, G. Enzymatic Purification of Microplastics in Environmental Samples. *Environ. Sci. Technol.* **2017**, *51* (24), 14283–14292. https://doi.org/10.1021/acs.est.7b03055.

(2) Kukulka, T.; Proskurowski, G.; Morét-Ferguson, S.; Meyer, D. W.; Law, K. L. The Effect of Wind Mixing on the Vertical Distribution of Buoyant Plastic Debris. *Geophys. Res. Lett.* **2012**, *39* (7). https://doi.org/10.1029/2012GL051116.

(3) Lebreton, L.; Slat, B.; Ferrari, F.; Sainte-Rose, B.; Aitken, J.; Marthouse, R.; Hajbane, S.; Cunsolo, S.; Schwarz, A.; Levivier, A.; Noble, K.; Debeljak, P.; Maral, H.; Schoeneich-Argent, R.; Brambini, R.; Reisser, J. Evidence That the Great Pacific Garbage Patch Is Rapidly Accumulating Plastic. *Sci. Rep.* **2018**, *8* (1), 1–15. https://doi.org/10.1038/s41598-018-22939-w.

(4) Eriksen, M.; Lebreton, L. C. M.; Carson, H. S.; Thiel, M.; Moore, C. J.; Borerro, J. C.; Galgani, F.; Ryan, P. G.; Reisser, J. Plastic Pollution in the World's Oceans: More than 5 Trillion Plastic Pieces Weighing over 250,000 Tons Afloat at Sea. *PLoS One* **2014**, *9* (12), 1–15. https://doi.org/10.1371/journal.pone.0111913.

(5) Pan, Z.; Sun, X.; Guo, H.; Cai, S.; Chen, H.; Wang, S.; Zhang, Y.; Lin, H.; Huang, J. Prevalence of Microplastic Pollution in the Northwestern Pacific Ocean. *Chemosphere* **2019**, *225*, 735–744. https://doi.org/10.1016/j.chemosphere.2019.03.076.

(6) Goldstein, M. C.; Titmus, A. J.; Ford, M. Scales of Spatial Heterogeneity of Plastic Marine Debris in the Northeast Pacific Ocean. *PLoS One* **2013**, *8* (11). https://doi.org/10.1371/journal.pone.0080020.

(7) Egger, M.; Quiros, L.; Leone, G.; Ferrari, F.; Boerger, C. M.; Tishler, M. Relative Abundance of Floating Plastic Debris and Neuston in the Eastern North Pacific Ocean. *Front. Mar. Sci.* **2021**, *8* (June), 1–13. https://doi.org/10.3389/fmars.2021.626026.
